# Four-stage dissolved oxygen strategy based on multi-scale analysis for improving spinosad yield by *Saccharopolyspora spinosa* ATCC49460

**DOI:** 10.1111/1751-7915.12264

**Published:** 2015-03-26

**Authors:** Yun Bai, Peng-Peng Zhou, Pei Fan, Yuan-Min Zhu, Yao Tong, Hong-bo Wang, Long-Jiang Yu

**Affiliations:** 1 Institute of Resource Biology and Biotechnology, Department of Biotechnology, College of Life Science and Technology, Huazhong University of Science and TechnologyWuhan, 430074, China; 2 Key Laboratory of Molecular Biophysics Ministry of Education, Huazhong University of Science and TechnologyWuhan, 430074, China

## Abstract

Dissolved oxygen (DO) is an important influencing factor in the process of aerobic microbial fermentation. Spinosad is an aerobic microbial-derived secondary metabolite. In our study, spinosad was used as an example to establish a DO strategy by multi-scale analysis, which included a reactor, cell and gene scales. We changed DO conditions that are related to the characteristics of cell metabolism (glucose consumption rate, biomass accumulation and spinosad production). Consequently, cell growth was promoted by maintaining DO at 40% in the first 24 h and subsequently increasing DO to 50% in 24 h to 96 h. In an in-depth analysis of the key enzyme genes (*gtt*, *spn* A, *spn* K and *spn* O), expression of spinosad and specific Adenosine Triphosphate (ATP), the spinosad yield was increased by regulating DO to 30% within 96 h to 192 h and then changing it to 25% in 192 h to 240 h. Under the four-phase DO strategy, spinosad yield increased by 652.1%, 326.1%, 546.8%, and 781.4% compared with the yield obtained under constant DO control at 50%, 40%, 30%, and 20% respectively. The proposed method provides a novel way to develop a precise DO strategy for fermentation.

## Introduction

Dissolved oxygen (DO) plays a significant role in aerobic fermentation (Xu and Zhong, 2011). Using a relatively high DO in the fermentation process would lead to high energy consumption; on the contrary, relatively low DO would negatively affect cell growth (Chen *et al*., 2012). Researchers (Xu *et al*., 2009; Wang *et al*., 2010; Song *et al*., 2013) have studied two or more stages of a DO strategy in aerobic fermentation processes. Such stages improved the corresponding product yield. However, other influencing factors, such as intracellular nucleotides and gene expression, also affect fermentation products (Xavier *et al*., 2007). Therefore, a multi-scale analysis should be involved in the study of a DO strategy in the fermentation process.

Spinosad, an aerobic microbial-derived secondary metabolite of soil actinomycete *Saccharopolyspora spinosa* (*S. spinosa*) (Mertz and Yao, 1990), is a mixture of spinosyns A and spinosyns D, which are two major components in *S. spinosa* fermentation products (Huang *et al*., 2009). As a highly effective targeted pesticide, spinosad was awarded the Presidential Green Chemistry Challenge Award in 1999 for its low environmental impact, low risk to non-target species and low mammalian toxicity (Sparks *et al*., 2001). Oxygen is an indispensable raw material that must be supplied in large amounts in industrial spinosad production. Luo and colleagues (2012) have integrated the expression of *Vitreoscilla* haemoglobin gene in *S. spinosa*, thereby improving oxygen uptake. Such integration was effective for the genetic improvement of *S. spinosa* fermentation. However, information on the integrated DO strategy for efficient spinosad production by *S. spinosa*-submerged fermentation is lacking. Fortunately, details of the spinosad biosynthesis pathway and the genome information of *S. spinosa* have been elucidated recently (Madduri *et al*., 2001; Hong *et al*., 2006; Huang *et al*., 2008; Yang *et al*., 2014). Many other microbial DO strategies were established successfully (Garcia-Ochoa *et al*., 2010; Cao *et al*., 2013; Huang *et al*., 2013). Those strategies were useful for the study of DO strategy on spinosad production.

In this paper, the reactor levels of DO conditions related to the characteristic of cell metabolism (spinosad yield, glucose consumption rate, biomass accumulation, and ATP production) and expressions of four key enzyme genes (*gtt*, *spn* A, *spn* K and *spn* O) for spinosad production were studied. We investigated DO in relation to the three scales mentioned above to establish a multi-stage DO control, reduce energy consumption, save costs and set up an efficient fermentation production process.

## Results

### Spinosad production at different DO levels

To investigate the effects of DO on spinosad production, different oxygen conditions were analysed at constant DO (20%, 30%, 40% and 50%). Any other fermentation conditions were not changed in these processes.

**Fig 1:**
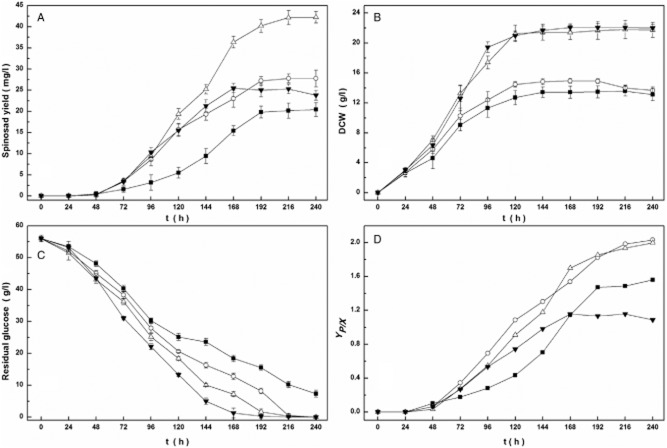
Spinosad production at different DO level of (■) 20%, (○) 30%, (△) 40% and (▼) 50%. Changes over time in (A) spinosad yield, (B) DCW, (C) residual glucose and (D) *Y_P_*_/_*_X_*. Each value is the mean of three parallel replicates.

As shown in Fig. 1, the glucose consumption rate was the highest before120 h and then decreased along with the glucose concentration at 50% DO, and almost no glucose was used after 168 h. When at 30% and 20% DO, cell growth and glucose consumption rate decreased obviously, at the same time the dry cell weight (DCW) were lower than that at 50% DO. The maximum DCW and maximum *Y_P/X_* (mg l^−1^ spinosad yield / g l^−1^ DCW), were achieved at 50% and 30% DO during 24–96 h respectively. The result indicates that the high DCW and *Y_P/X_* could not be achieved simultaneously by controlling a constant DO during the whole culture process. This process could be at least divided into two stages, which are cell growth and product synthesis. Accordingly, cell growth at 50% DO and spinosad production at 30% DO are likely to be optimal. Furthermore, the DCW under 50% DO was not significantly different with that under 40% DO before 24 h, but during 24–96 h the former (DCW 19.43 ± 0.70) became higher than the latter (DCW17.40 ± 0.85) (*P* < 0.05). In consideration of power saving, maintaining 40% DO at the first 24 h and then changing it to 50% from 24 h to 96 h could be a better choice. In order to realize a more precisely DO-controlling spinosad production process, a further investigation should be conducted.

Based on above experimental results, we can ensure 0–24 h DO 40%, 24–96 h DO 50%, but DO is undetermined yet at 96–240 h. According to the Table 2 results, the different three-phase DO strategy were studied. Table 2 shows the 35%, 30%, 25%, 20% DO, respectively, during 96–240 h; the DCW and spinosad yield were no difference between 25% and 30% DO at 96–240 h; the spinosad yield at 35%; and 20% DO were lower than 30% and 25% DO. The results show that it is beneficial for spinosad production when DO is controlled at 25% or 30% at 96–240 h. To detect the optimal DO level during 96–240 h of the fermentation process, we need in-depth research.

**Table 1:** Comparison of spinosad production at different three-phase DO strategy

Different DO strategy	DCW(g l^−1^)	Spinosad yield (mg l^−1^)	Residul glucose (g l^−1^)	*Y_P/X_*
40%^a^–50%^b^–35%^c^	21.82 ± 0.10	80 ± 1.01	0 ± 0.10	3.67
40%^a^–50%^b^–30%^c^	22.00 ± 0.11	138 ± 1.23	0 ± 0.12	6.27
40%^a^–50%^b^–25%^c^	22.02 ± 0.10	135 ± 1.32	0 ± 0.11	6.13
40%^a^–50%^b^–20%^c^	21.20 ± 0.15	91 ± 1.77	0 ± 0.13	4.26

a Means fermentation during 0–24 h.

b Means fermentation during 24–96 h.

c Means fermentation during 96–240 h.

### Key enzyme gene expression under different three-phase DO strategy

*Spn* A, *spn* K *spn* O and *gtt* involved in spinosad biosynthesis pathway were selected as target genes in this research because these four genes encode rate limited enzymes in charge of spinosad biosynthesis (Xue *et al*., 2013a). The expression of selected genes involved in spinosad biosynthesis was monitored using quantitative reverse transcriptase polymerase chain reaction (QRT-PCR) to investigate how DO influences spinosad production. The expression ratios of all these target genes were compared by 2^−ΔΔCT^ method.

**Fig 2:**
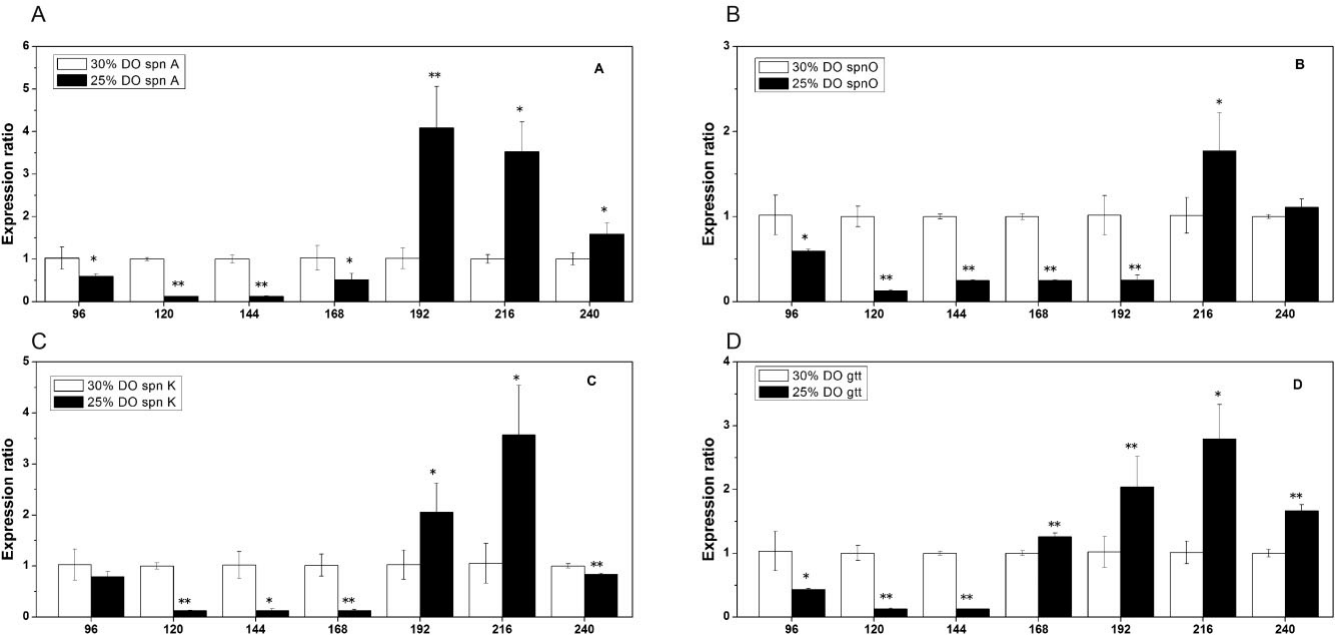
Relative expression ratios (experimental group_(40%–50%–25%__DO__)_/control group_(40%–50%–30%__DO__)_) of four rate-limited spinosad biosynthesis genes – (A) *spn* A, (B) *spn* K, (C) *spn* O and (D) *gtt* – at 96, 120, 144, 168, 192, 216 and 240 h. The *P < 0.05 and **P < 0.01 were obtained from the *t*-test for statistical analysis.

As shown in Fig. 2, the transcript levels of *spn* A (Fig. 2A), *spn* K (Fig. 2B), *spn* O (Fig. 2C) and *gtt* (Fig. 2D) under 30% DO significantly increased compared with 25% DO in 96 h–168 h. The transcript levels of *spn* A (Fig. 2A), *spn* K (Fig. 2B), *spn* O (Fig. 2C) and *gtt* (Fig. 2D) under 25% DO significantly increased compared with 30% DO in 168–240 h. In Fig. 2, we illustrate that it is more conducive to the accumulation of spinosad 30% DO in 96–168 h and with 25% DO in 192–240 h.

### The SATP levels with different three-phase DO strategy

Table 2 shows that the DCW and spinosad yield at 30% DO were not significantly different with that at 25% DO. But the expression ratios of key enzyme genes were significantly different (Fig. 2). Generally, traditional techniques for quantifying biomass are not able to provide information about the quality, activity or viability of the microorganisms. Nevertheless, SATP (mg ATP g^−1^ DCW) can be as a biomass quality indicator (Gikas and Livingston, 1998).

**Fig 3:**
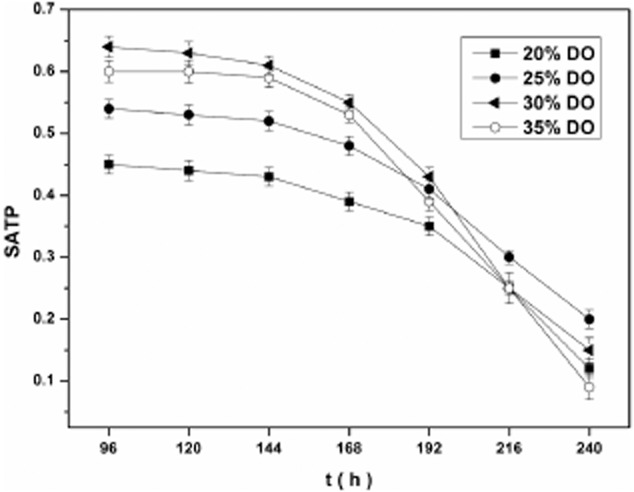
The comparison of SATP among 20%, 25%, 30% and 35% DO strategy after 96 h, which DO are all under 40% during 0–24 h and 50% during 24–96 h.

The SATP between different three-phase DO strategies (20%, 25%, 30% and 35% DO during 96–240 h) were shown in Fig. 3. Compared with other DO conditions, SATP level was the maximum under 30% DO during 96–192 h, after when it dropped more rapidly than the others. In the meantime, under 25% Do, the SATP level was slowly reduced. The result shows that at the beginning of the stationary phase, 30% DO is better than 25% DO, and in the later period of the stationary phase, it is proper to regulate DO to a lower level for maintaining cell vitality.

### Four-phase DO strategy for spinosad production

**Fig 4:**
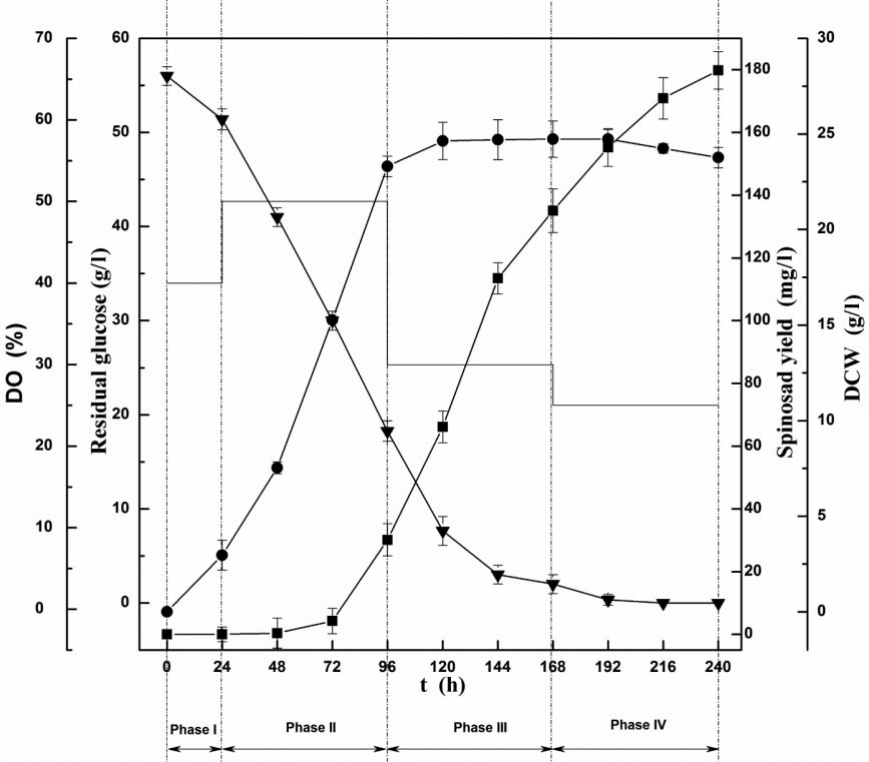
Changes over time in spinosad fermentation with four-stage DO strategy in a 10-l bioreactor. (■) Spinosad yield, (●) DCW, (▼) residual glucose and (^____^) DO. The error bars indicate the standard deviations of three independent samples.

According to above results, a four-phase DO strategy was investigated by controlling DO at 40% during 0–24 h, 50% during 24–96 h, 30% during 96–192 h, 25% until the end of the fermentation. Spinosad fermentation in 10 l of bioreactors using four-phase DO strategy was shown in Fig. 4. Each phase corresponds to the particular physiological characteristics of spinosad biosynthesis. At Phase I (0–24 h) with DO 40%, the nutrients were used for cell growth. At Phase II (24–96 h) with DO 50%, the nutrients were used for cell growth and a small quantity of spinosad was produced. At Phase III (96–192 h) with DO 30%, cell growth went into stationary state, and spinosad production rate increased rapidly. At the Phase IV (192–240 h), spinosad production was still in high rate by adjusting DO to 25%. The result of the four-phase DO control shows that spinosad yield was 179.8 ± 8.0 mg l^−1^, which is increased by 652.1%, 326.1%, 546.8% and 781.4%, compared with constant DO control at 50%, 40%, 30% and 20% respectively.

## Discussion

DO affects cell growth and product biosynthesis in numerous microorganisms (Li *et al*., 2012; Chang *et al*., 2014). An excessive oxygen supply would decrease productivity because of the direct aerobic respiration of substrates, thereby dramatically elevating the costs of production in industrial fermentation (Käß *et al*., 2014). However, a relatively low DO would negatively affect cell growth. A successful industrial fermentation process involves higher productivity and a cost-effective production strategy. Cell growth of *S. spinosa* ATCC49460 depends on the TCA cycle (Yang *et al*., 2014), in which DO concentration needs to remain at a high level. Spinosad production begins to increase at the start of the stationary phase of *S. spinosa* ATCC49460 growth. This phase does not require a high DO concentration, but the synthesis of spinosyns A (C_41_H_65_NO_10_) and D (C_42_H_67_NO_10_) requires oxygen. Accordingly, the DO needs to remain at a proper level. DO affects cell growth and spinosad biosynthesis, and a higher concentration of DO is required for the former than for the latter. A two-stage oxygen supply strategy can ensure spinosad production by *S. spinosa* ATCC 49460. Researchers (Jin *et al*., 2008; Xu *et al*., 2009; Rosa *et al*., 2010) used a similar method to establish a two-phase DO strategy.

A DO strategy can be established by determining the DO tension that is related to biomass and productive yield. However, other influencing factors, such as intracellular nucleotides and gene expression, also affect the fermentation products (Zhong *et al*., 2004). In our study, spinosyns were used to establish a DO strategy by multi-scale analysis, which includes reactor, cell and gene scales. Similar results were obtained by Liang and colleagues (2004) who presented an online data association analysis performed on multiple scales and a fermentation regulation method that was based on the analysis and control of the metabolic flux.

The spinosad biosynthetic genes are 80 kb (Waldron *et al*., 2000), including five large genes (*spn* A, B, C, D and E) encoding type I polyketide synthase, four genes (*spn* F, J, L and M) involved in intramolecular C–C bond formation, four genes (*spn* G, I, K and H) involved in rhamnose attachment and methylation and six genes (*spn* P, O, N, Q, R and S) involved in forosamine biosynthesis. The genes for synthesis of rhamnose (*gtt*, *gdh*, *epi* and *kre*) are not closely linked to the spinosyn gene cluster (Waldron *et al*., 2001; Hong *et al*., 2006; 2008). Four key enzyme genes (*gtt*, *spn* A, *spn* K and *spn* O) for spinosad production were studied, and the results demonstrated how DO changes affect spinosad production. Similar results were obtained by Xue and colleagues (2013b), who compared the expression of four key enzyme gene (*gtt*, *spn* A, *spn* K and *spn* O) at 120, 144, 168, 192, 216 and 240 h with and without additional exogenous fatty acids. Results elucidated why the change of exogenous fatty acids could affect spinosad production.

Bacterial viability is difficult to define accurately by DCW. Thus, biomass activity was determined to measure the ability of biomass to metabolize a particular substrate, thereby describing the potential activity of biomass. SATP has been frequently used as a biomass quality indicator because ATP is present in all living organisms and is involved in most biochemical pathways. The result in Fig. 3 illustrates the slowing down of cell metabolism toward the end of the stability phase and the decrease in oxygen demand. If DO remained at a high level, reduced cell vitality and the production of spinosad would be observed. Similar results were reported by Zhang and colleagues (2012), who found that curdlan production had a positive relationship with intracellular levels. Thus, a simple and reproducible two-stage DO control process was developed.

Optimizing the industrial production of spinosad by *S. spinosa* is difficult because large numbers of physiological regulatory mechanisms occur during the process. A holistic concept cannot be obtained by just a single reactor regulatory factor. In our study, spinosyns were used to establish a DO strategy by multi-scale analysis, which included reactor, cell and gene scales. The characteristics of cell metabolism under different DO conditions were investigated. Oxygen demand was higher before the stationary phase, during which cell growth and glucose consumption rate were rapid. Starting from the late logarithmic phase of *S. spinosa* ATCC49460, synthesis of a large amount of spinosad began, whereas the demand for DO and the glucose consumption rate started to decrease. This finding indicated that the process could be divided into two stages, namely, cell growth and spinosad synthesis. Subsequently, in an in-depth analysis of the key enzymes gene expression of spinosad synthesis and SATP, the expressions of four key enzyme genes (*gtt*, *spn* A, *spn* K and *spn* O) resulted in high DO, which could lead to negative spinosad biosynthesis. The SATP data showed that cell metabolism slowed toward the end of the stability phase and oxygen demand decreased. A more precise oxygen supply strategy can be ensured for spinosad synthesis. Finally, a four-phase DO strategy was established.

To our knowledge, this is the first report on the effects of various DO concentrations combined with multi-scale analysis on spinosad fermentation. Our results would help elucidate spinosad production and metabolic response of *S. spinosa* ATCC49460 under various DO conditions. Furthermore, applying the proposed four-stage DO strategy on fermentation models would help develop and improve the production processes of other aerobic microbial-derived secondary metabolites.

## Experimental procedures

### Microorganism and medium

#### Microorganism: S. spinosa *ATCC49460*

The plate medium was composed of the following (g l^−1^): glucose, 10.0; N-Z Amine Type A, 2.0; yeast extract, 1.0; and agar, 15.0, and incubated at 30°C up to 8 days. The spores from the plate culture were inoculated into a 250 ml Erlenmeyer flask containing 25 ml of seed medium. The seed medium was composed of the following (g l^−1^): glucose, 10.0; N-Z Amine Type A, 2.0; yeast extract, 1.0; K_2_HPO_4_, 0.2; and MgSO_4_, 2.0; the pH was adjusted to 7.0 before autoclaving. After incubation at 30°C on a rotary shaker at 220 r.p.m. for 60 h, a 2 ml portion of the seed culture was used to inoculate 25 ml of production medium into a 250 ml Erlenmeyer flask fermentation medium. The fermentation medium was composed of the following (g l^−1^): glucose, 60.0; cottonseed protein, 20.0; yeast extract, 8.0; CaCO_3_, 1.0; and rapeseed oil, 10.0. In all cases, the medium was sterilized using an autoclave for 20 min at 121°C. The flasks were incubated for 60 h on an orbital shaker (Infors Zhi Cheng, Shanghai, China) at 200 r.p.m. and 30°C. Approximately, 10% (v/v) of the seed culture was inoculated into the 10 l bioreactor. The culture was incubated for 240 h at 30°C.

### Fermentation in a 10 l bioreactor

The oxygen supply was also analysed during batch fermentation in a 10 l bioreactor (Biotech-10JS, Shanghai, China) with 7 l working volume. The DO probe (Mettler-Toledo GmbH, Switzerland) and pH probe (Mettler-Toledo GmbH, Switzerland) measured the DO, pH and temperature, agitation speed were measured online. The pH was adjusted using 2.0 mol l^−1^ NaOH or 2.0 mol l^−1^ HCl. The temperature was controlled automatically. Different constant DO 20%, 30%, 40% and 50% in 10 l bioreactors were studied. The three-stage controls were performed as follows: during 0–24 h, the DO was set to 40%; during 24–96 h, the DO was set to 50%; and during 96–240 h, the DO was set to 30%. The four-stage oxygen controls were performed as follows: at Phase I (0–24 h), the DO was set to 40%; at the subsequent culture Phase II (24–96 h), the DO was set to 50%; at Phase III (96–192 h), the DO was set to 30%; at Phase IV (192–240 h), the DO was set to 25%. The DO level (percentage of air saturation) was controlled at constant values (20%, 30%, 40% and 50%) or varying values by cascading different agitation speeds (from 150 up to 500 r.p.m.), and the ventilation was 1 vvm throughout. The other culture conditions were the same as in the above experiments. Three batches were repeated for each experiment.

### Determinations

The spinosad was determined as described by Yáñez and colleagues (2014).

To determine the DCW, 10 ml of the fermentation broth was centrifuged at 8000 × *g* for 10 min, and the supernatant was discarded. After washing twice with distilled water, the supernatant was discarded and the cell precipitate was dried to a constant weight at 60°C for 48 h. All cell pellets were weighed after drying. The glucose concentration was measured using the 3,5-dinitrosalicylic acid spectrometric method (Miller, 1959).

Extraction and determination of the intracellular ATP levels: the concentrations of intracellular ATP were determined by an in vitro procedure based on inactivation of metabolism, which method according to the method described by Dhople and Hanks (1973). All experimental values were presented as the means of three replicates ± standard deviation.

### RNA extraction, cDNA synthesis and qPCR

The method of RNA extraction, cDNA synthesis and qPCR according to the method (Song *et al*., 2014) described with some modifications. Total RNA were isolated from the samples at 96, 120, 144, 168,192, 216 and 240 h. For each time point, three biological replicates were obtained and analysed. Specific primers were designed for genes involved in spinosad biosynthesis (Table 1 lists the primers used in this study). The internal control used was 16S rRNA. 16S rRNA was a suitable reference gene in this study, which has been determined in another study. The data obtained were analysed by applying the 2^−ΔΔCT^ method, −ΔΔC_T_ = (C_T(trager gene)_-C_T(reference gene)_)_test_ − (C_T(trager gene)_-C_T(reference gene)_)_calibrator_, where C_T_ is the threshold cycle number of the target gene at any collection time, C_T(reference gene)_ is the expression quantity of 16S rRNA at the same collection time as Ct, (C_T(trager gene)_-C_T(reference gene)_)_test_ is the expression quantity of the target gene on day 25% DO and (C_T(trager gene)_-C_T(reference gene)_)_calibrator_ is the expression quantity of the target gene on day 30% DO. Statistical analyses were performed using Microsoft Excel (2007). All experiments were repeated three times.

**Table 2:** List of gene and gene primers used in this study

Gene name	Gene function	Primers	Primers Sequence 5′ → 3′
*gtt*	Rhamnose synthesis and cell synthesis	*gtt*-F	ACTTCCGGTGTACGACAAGC
		*gtt*-R	AAGCCCTGCCCGTAAAAGAT
*spn* A	Polyketide synthesis	*spn* A-F	TTGGTACGCCACACTGTCTC
		*spn* A-R	GAGCGCATCCAGATAGGCAT
*spn* K	Rhamnose methylation	*spn* K-F	GTCAGGACGAAGTCAACGGT
		*spn* K-R	ATGTCCACAACGCACGAGAT
*spn* S	Forosamine synthesis	*spn* S-F	GGAAACCACCAGGGTTCGAT
		*spn* S-R	ATTCGCAGAAACCTCCTCGG
16S rRNA	Conserved sequence	16S rRNA-F	CCTACGAGCTCTTTACGCCC
		16S rRNA-R	AGAAGCACCGGCTAACTACG

## Conflict of interest

None declared.
